# Role of Long Non-Coding RNA LINC00641 in Cancer

**DOI:** 10.3389/fonc.2021.829137

**Published:** 2022-01-27

**Authors:** Xue Han, Shitai Zhang

**Affiliations:** Department of Obstetrics and Gynecology, Shengjing Hospital of China Medical University, Shenyang, China

**Keywords:** lncRNA - long noncoding RNA, LINC00641, cancer, biomarker, therapeutic target

## Abstract

Long non-coding RNAs (lncRNAs) are non-protein coding RNAs with more than 200 nucleic acids in length. When lncRNAs are located in the nucleus, they regulate chromosome structure, participate in chromatin remodeling, and act as transcription regulators. When lncRNAs are exported to the cytoplasm, they regulate mRNA stability, regulate translation, and interfere with post-translational modification. In recent years, more and more evidences have shown that lncRNA can regulate the biological processes of tumor proliferation, apoptosis, invasion and metastasis, and can participate in a variety of tumor signaling pathways. Long-gene non-protein coding RNA641 (LINC00641), located on human chromosome 14q11.2, is differentially expressed in a variety of tumors and is related to overall survival and prognosis, etc. Interfering the expression of LINC00641 can lead to changes in tumor cell proliferation, invasion, metastasis, apoptosis and other biological behaviors. Therefore, LINC00641 is a promising new biomarker and potential clinical therapeutic target. In this review, the biological functions, related mechanisms and clinical significance of LINC00641 in many human cancers are described in detail.

## Background and Introduction

Cancer is a global and growing disease, which still has high incidence rate and mortality rate. For the exploration of cancer, from genomics (1), epigenetics (2), to tumor microenvironment (3), immunotherapy (4), to cancer cell metabolism (5) and tumor microbiota (6), people have never stopped moving forward.

**Figure 1:**
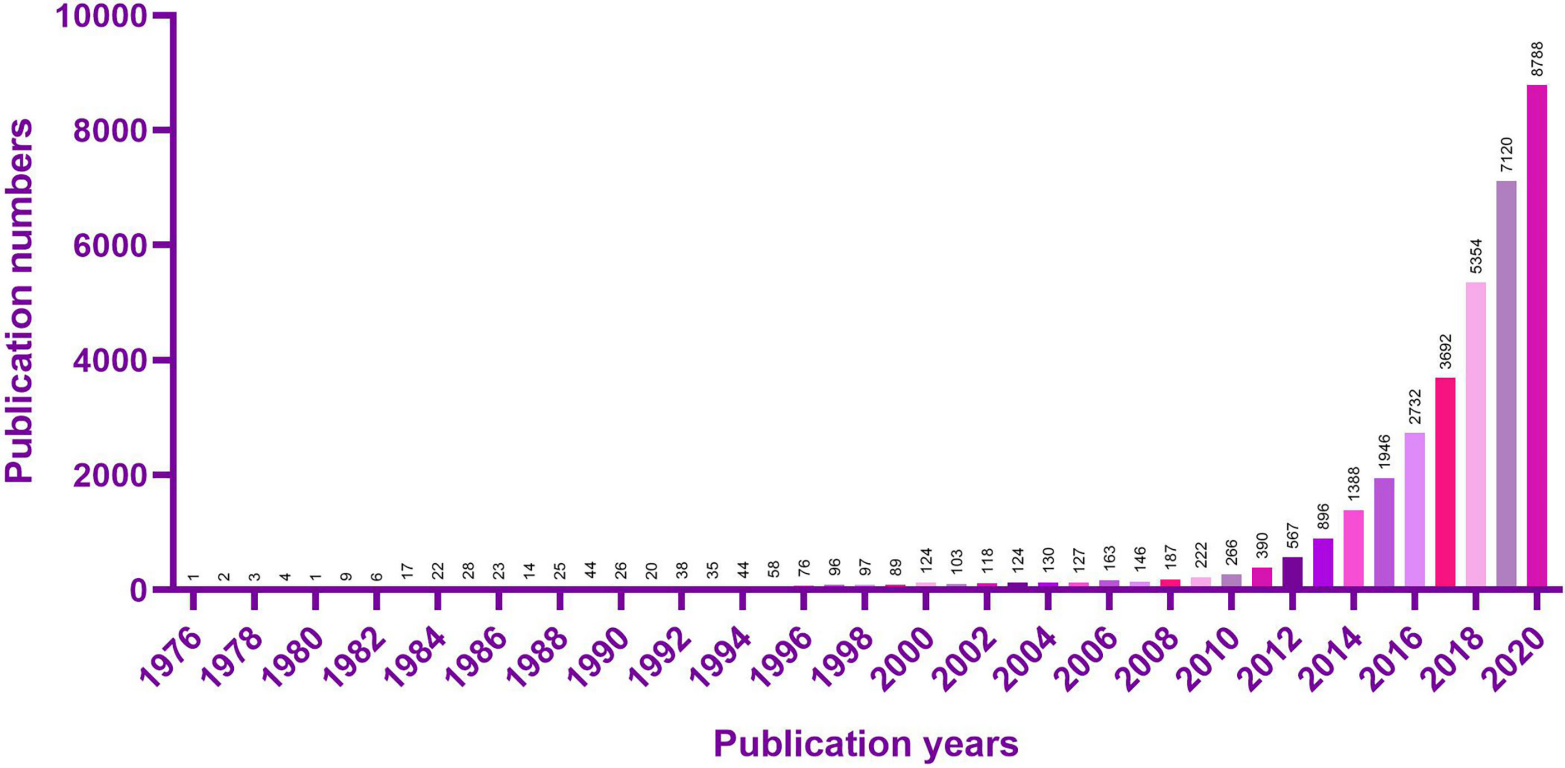
PubMed search results using the keyword “LncRNA” are displayed as a histogram.

Non protein coding genes, which account for about 98% of the total genome sequence (7), have long been removed from the transcriptional “noise”. Long non-coding RNA (LncRNA) with a length of more than 200 nucleotides (8) can participate in the regulation of gene expression at epigenetic (9), transcriptional and post transcriptional levels (10), adopting the action mode of signal, bait, guide or scaffold (11). The researches on LncRNA have sprung up in recent ten years (**Figure 1**).

LncRNAs perform different functions according to their localization in subcells (12). When LncRNA located in the nucleus, it regulates chromosome structure (13), participates in chromatin remodeling (14), and acts as a transcription regulator (15). When lncRNA is exported to the cytoplasm, it regulates mRNA stability (16), regulates translation (17), and interferes with post-translational modifications (18).

Mutation and misregulation of long non-coding RNA (LncRNA) play an important role in cancer (19), and participate in multiple biological processes such as tumor proliferation, apoptosis, invasion and metastasis (20–23). LncRNA can participate in a variety of tumor signaling pathways, including p53 (24) and NF-κB (25)、PI3K/AKT (26)、 Wnt/β- Catenin (27) and notch (28), etc. The most important feature of LncRNA mediated cell signal regulation is that LncRNA can act as a scaffold (29). A large number of papers have identified many LncRNAs, which can regulate gene expression in various cancers through ceRNA regulation. LncRNA can be used as a “sponge” to adsorb microRNA, blocking the inhibition of microRNA on its downstream target mRNA, and indirectly regulating the expression of genes, which functions as a microRNA decoy (30–33).

**Figure 2:**
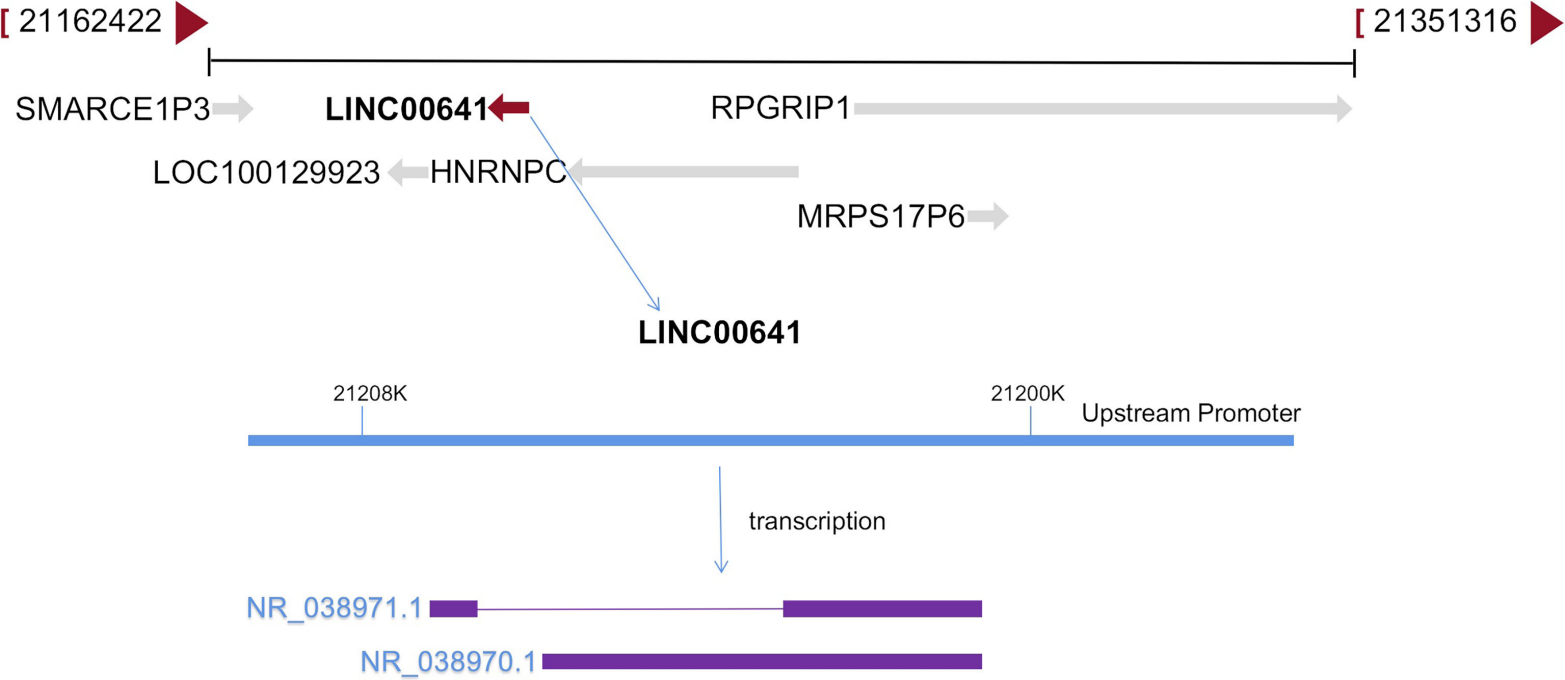
LINC00641 formation diagram.

**Figure 3:**
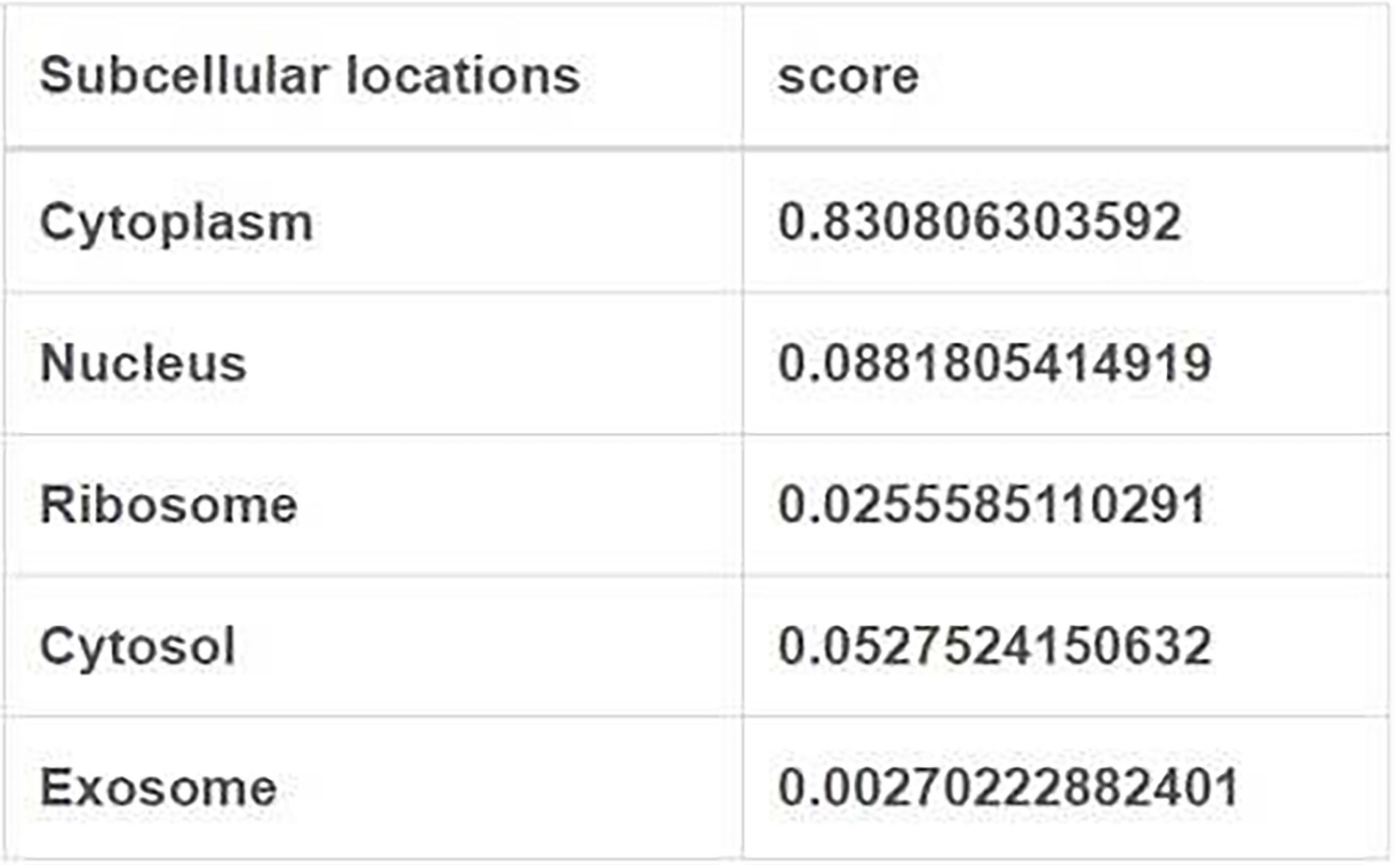
Subcellular localization of LINC00641 (from lnlocator database).

The long intergenic non protein coding RNA641 (LINC00641) is located on human chromosome 14q11.2. It is widely expressed in 22 tissues such as brain tissue and bone marrow, among which the expression in brain tissue is the highest (34). More and more evidence show that LINC00641 is differentially expressed in a variety of tumors and is associated with overall survival and prognosis (35–37). LINC00641 can participate in the regulation of proliferation, invasion, metastasis and apoptosis of a variety of tumors (38–40). A search of NCBI database revealed that LINC00641 gene sequence could be transcribed into two transcripts: NR_038970.1 and NR_038971.1 (**Figure 2**). According to the lnclocator database, LINC00641 is mainly located in the cytoplasm (**Figure 3**).

LINC00641 is downregulated in bladder cancer, cervical cancer, breast cancer, prostate cancer, non-small cell lung cancer, glioma and cutaneous squamous cell carcinoma, and can be used as a tumor suppressor gene, while it is up-regulated in acute myeloid leukemia, gastric cancer, rectal cancer and renal cell carcinoma, and can be used as oncogene. Considering the different expression of LINC00641 in different tumors, it may be related to tissue specificity. In this paper, we elaborate the biological function, related mechanism and clinical significance of LINC00641 in human cancer (**Table 1**).

**Table 1:** Functional characteristics and clinical significance of LINC00641 in different cancers.

Cancer	Expression	Function	Related gene	Role	Clinical significance	Reference
Bladder cancer	Down regulation	Proliferation, migration, invasion	miR-197-3p、KLF10	Tumor suppressor gene	Overall survival and progression free survival	(35)
Cervical cancer	Down regulation	Proliferation, migration, invasion, apoptosis	miR-378a-3p、CPEB3	Tumor suppressor gene		(41)
Breast cancer	Down regulation	Proliferation, migration, invasion, apoptosis, cell cycle	miR-194-5p	Tumor suppressor gene	Tumor size, lymph node metastasis and clinical stage	(42)
Prostatic cancer	Down regulation	Proliferation, invasion, apoptosis	miR-365a-3p、VGLL4	Tumor suppressor gene	Overall survival	(36)
Non small cell lung cancer	Down regulation	Proliferation, apoptosis, migration	miR-424-5p、PLSCR4	Tumor suppressor gene		(39)
Glioma	Down regulation	Proliferation, apoptosis	miR-4262、NRGN	Tumor suppressor gene		(43)
Cutaneous squamous cell carcinoma	Down regulation	Proliferation, migration, invasion	miR-424	Tumor suppressor gene		(44)
Acute myeloid leukemia	Up regulation	Proliferation, migration, invasion, apoptosis, cell cycle	miR-378a、ZBTB20	Oncogene		(40)
Gastric cancer	Up regulation	Proliferation, migration, autophagy, drug resistance, invasion, apoptosis	miR-582-5p、miR-429、Notch-1	Oncogene	Overall survival, Oxaliplatin resistance, M stage and patient gender	(38, 45)
Colorectal cancer	Up regulation	Proliferation, migration	miRNA-424-5p、PLSCR4	Oncogene	Overall survival	(46)
Renal cell carcinoma	Up regulation	Proliferation, invasion, apoptosis	microRNA-340-5p	Oncogene	Tumor stage and overall survival	(37)

## Role of LINC00641 in Cancer

### Bladder Cancer

Bladder cancer is a common malignant tumor in women and the fourth most common malignancy tumor in men (47). Worldwide, there are an estimated 500,000 new cases and 200,000 deaths each year (48). Research into cancer genomics, risk factors and immunotherapy is the key to combat this malignant disease (49). It is very important to explore the molecular mechanism of bladder cancer development. Li et al. (35) analyzed the expression of LINC00641 in 39 pairs of bladder cancer tissues and normal tissues and found that LINC00641 was significantly decreased in bladder cancer tissues, and the low expression of LINC00641 was associated with overall survival and progression-free survival of patients. *In vitro*, the overexpression of LINC00641 reduced the proliferation, migration and invasion of bladder cancer cell lines. *In vivo*, LINC00641 overexpression significantly inhibited tumor volume. Starbase bioinformatics analysis showed that LINC00641 may interact with miR-197-3p and using Targetscan 7 tool, it was determined that miR-197-3p may target KLF10. The results showed that increasing the expression of LINC00641 inhibited miR-197-3p, while miR-197-3p inhibited the expression of KLF10 in cells. In a myeloma study, KLF10 inactivated the PTEN/PI3K/AKT pathway. The experiment also demonstrated that LINC00641 promoted KLF10 expression by targeting miR-197-3p, thereby inhibiting PTEN/PI3K/AKT pathway. In conclusion, LINC00641 can competitively bind to miR-197-3p through the ceRNA mechanism to increase the expression of KLF10, further inhibit the activation of PTEN/PI3K/AKT pathway, and inhibit the proliferation, migration and invasion of bladder cancer cells. In general, LINC00641 is a tumor suppressor factor in bladder cancer. LINC00641/miR-197-3p/KLF10/PTEN/PI3K/AKT cascade may be a promising target for bladder cancer treatment.

### Cervical Cancer

Cervical cancer is the fourth most common cancer among women worldwide, with more than 85% of cervical cancer deaths occurring in less developed regions of the world (50). Although HPV vaccine is one of the major breakthroughs in modern medicine, and much is known about HPV induced carcinogenesis, the clinical outcomes have been stagnated for decades (51). More and more cervical cancer genome analyses have revealed new potential therapeutic targets for cervical cancer (52). By studying the molecular mechanism of cervical cancer, we aim to provide some methods for targeted therapy. Zhang et al. (41) found that the expression of LINC00641 was significantly down regulated in cervical cancer cell lines compared with normal cervical cells. Upregulation of LINC00641 inhibited cell proliferation, increased apoptosis, decreased invasion and migration, and inhibited EMT. Fish showed that LINC00641 was abundant in the cytoplasm of cervical cancer cells. After further exploring the mechanism of LINC00641, combined with Starbase bioinformatic analysis and experiments, it was proved that miR-378a-3p was the target of LINC00641 and LINC00641 inhibited the growth of cervical cancer cells by reducing the expression of miR-378a-3p. Starbase database was used to predict the opposite changes of CPEB3 and miR-378a-3p. The experiment proved that LINC00641 competed with CPEB3 and bound to miR-378a-3p, confirming that LINC00641 regulated the progression of cervical cancer by up regulating CPEB3. In conclusion, LINC00641 can competitively bind miR-378a-3p through the ceRNA mechanism, so as to increase the expression of CPEB3 mRNA and inhibit the proliferation, migration and invasion of cervical cancer cells. In general, LINC00641 is a tumor suppressor of cervical cancer and plays an important role in its occurrence and development. LINC00641 deserves to be regarded as a new biomarker in the treatment of cervical cancer.

### Breast Cancer

Breast cancer is the most frequently diagnosed cancer among women, ranking the second among the causes of cancer-related deaths in women (53). Although the use of adjuvant chemotherapy, the widespread use of hormonal drugs and the application of targeted drugs have reduced the mortality of breast cancer, biomarkers are still an important method for assisting diagnosis and monitoring prognosis. The American Society of Clinical Oncology once recommended CA 15-3, carcinoembryonic antigen, estrogen and progesterone receptor for clinical diagnosis (54). For the treatment of breast cancer, molecular mechanism and potential therapeutic target have become a research hotspot (55). Mao et al. (42) found that LINC00641 expression in breast cancer tissues was significantly lower than that in normal adjacent tissues, and was significantly correlated with tumor size, lymph node metastasis and clinical stage. Over expression of LINC00641 inhibits cell proliferation, migration, invasion and G1/S phase transition and promotes cell apoptosis in breast cancer cell lines. According to starbase 3 and miRcode analysis, miR-194-5p may bind to LINC00641. Previous studies (56) have found that miR-194-5p can promote the growth and metastasis of breast cancer cells and act as an oncogene. It is confirmed that miR-194-5p is a direct target of LINC00641 and the overexpression of miR-194-5p can reverse the inhibition of LINC00641 up-regulation on the biological behavior of breast cancer cells. Therefore, the molecular mechanism of LINC00641 overexpression inhibiting proliferation, migration and invasion of breast cancer cells can be elucidated through the sponge action of miR-194-5p, and LINC00641/miR-194-5p may contribute to the treatment of breast cancer as a favorable target.

### Prostatic Cancer

Prostate cancer is the major disease affecting men’s health all over the world and is the second most common form of cancer in men, second only to non-melanoma skin cancer (57). Since Wang et al. first purified the protein in 1979, the diagnostic strategy of population screening for prostate cancer using prostate specific antigen (PSA) has been controversial and hotly debated (58). At present, the treatment of prostate cancer patients increases the demand for reliable biomarkers and pursues individual patient-centered oncology approach (59). Liu et al. (36) found that the expression of LINC00641 was down regulated in prostate cancer tissues and correlated with the prognosis of patients after comparing prostate cancer tissues with normal tissues. Overexpression of LINC00641 inhibited cell proliferation, invasion and promotes apoptosis in prostate cancer cell lines. When further exploring the mechanism of LINC00641 in prostate cancer, it was found that miR-365a-3p was a downstream target of LINC00641 and miR-365a-3p was up-regulated in prostate cancer and negatively correlated with LINC00641. Then it was found that VGLL4, as the downstream target gene of miR-365a-3p, was down regulated in prostate cancer and negatively correlated with miR-365a-3p. *In vitro* experiments demonstrated that LINC00641 regulates cell proliferation and invasion through miR-365a-3p/VGLL4 axis. In conclusion, LINC00641 can be used as a tumor suppressor of prostate cancer, which can increase the expression of VGLL4 and inhibit the proliferation and invasion of prostate cancer cells by competitively binding miR-365a-3p through the ceRNA mechanism. Huang et al. (60) retrieved prostate cancer tissues and normal tissues from the Cancer Genome Atlas (TCGA) database, screened 14083 lncRNAs, and finally identified 6 lncRNAs with independent prognostic factors, including LINC00641. A risk scoring model was established to effectively evaluate the survival and prognosis of patients. These results indicated that lncRNA has certain predictive effect on the occurrence and prognosis of prostate cancer and can be used as a new biomarker for prostate cancer survival and potential therapeutic targets.

### Non Small Cell Lung Cancer

Lung cancer is the most common cause of cancer death worldwide, with an estimated 1.6 million deaths per year (61), and non-small cell lung cancer(NSCLC) accounts for about 85%. Over the past two decades, important advances have been made in the treatment of NSCLC, increasing our understanding of disease biology and tumor progression mechanism, and promoted early detection (62). As a biomarker, lncRNA can be combined with traditional tumor markers such as CEA, SCCA and CYFRA21-1 in the diagnosis of NSCLC with great potential (63). Li et al. (39) found that LINC00641 expression was significantly reduced in non-small cell lung cancer cell lines compared with human normal bronchial epithelial cells. Overexpression of LINC00641 in the cell line decreased the ability of proliferation and migration and increased apoptosis. Starbase database predicted that miR-424-5p was a downstream target of LINC00641, further demonstrating that miR-424-5p could be directly bind to LINC00641 and was negatively regulated by LINC00641. The TargetScan database predicted PLSCR4 as a candidate target of miR-424-5p. Experiments proved that LINC00641, as the ceRNA of PLSCR4, increased the expression of PLSCR4 by adsorbing miR-424-5p. Therefore, LINC00641 as a tumor suppressor may play a role in the treatment of non-small cell lung cancer. But there is also the opposite voice. Dong et al. (64) comprehensively analyzed the expression of mRNAs, lncRNAs and miRNAs by using the data of 509 lung adenocarcinoma, 473 lung squamous cell carcinoma tissues and 49 adjacent non-cancerous lung tissues in TCGA database and constructed the ceRNA network mechanism of lncRNA-miRNA-mRNA. It is predicted that LINC00641, as an oncogene, can competitively combine miR-1285-3p to regulate the expression of downstream target genes PIGA, AHCYL1 and ATP1B2, or combine miR-6860 to regulate the expression of MAP3K3, SHRK004M and FGD3, so as to affect the progression of lung cancer. Comparing the two literatures, the former expounds the antitumor effect of LINC00641 in non-small cell lung cancer at the cellular level but lacks tissue validation and *in vivo* experiments. While the latter predicts LINC00641 as an oncogene in lung adenocarcinoma and squamous cell carcinoma, but it is only a biological information prediction and lacks experimental evidence. In view of the opposite results in the two literatures, only by further expanding the sample size and perfecting the histological verification can we obtain a more accurate mechanism of LINC00641 in non-small cell lung cancer.

### Glioma

Glioma is the most common primary intracranial tumor, accounting for 81% of malignant brain tumors, which can lead to significant mortality and morbidity (65). Progress and reports have been made in the treatment of glioma from aspects of molecular genetics (66), epigenetics (67) and cell metabolism (68). As for silent messenger lncRNAs, based on the increasing number of functional lncRNAs abnormally expressed in glioma tissues and cell lines, lncRNAs may be crucial for the occurrence, progression and other malignant phenotypes of gliomas (69). Yang et al. (43) found that the expression of LINC00641 decreased significantly in glioma and cell lines and overexpression of LINC00641 in glioma cells inhibited cell proliferation and promoted apoptosis. When further exploring the mechanism of LINC00641, it was found that the expression of NRGN was down-regulated in glioma and positively correlated with LINC00641. According to the lnclocator database, LINC00641 is mainly located in the cytoplasm, and the regulation mechanism of lncRNA in the cytoplasm is mostly ceRNA. Combined with Starbase v3.0 prediction, miR-4262 was found to bind to both LINC00641 and NRGN. Further experiments demonstrated that LINC00641 enhanced the expression of NRGN in glioma cells through the absorption of miR-4262. In conclusion, LINC00641 competitively binds miR-4262 through the ceRNA mechanism to release NRGN, which plays an anti-tumor role in the progression of glioma, providing a new therapeutic target for glioma patients. Zhang et al. (70) obtained clinical information from the Chinese glioma Genome Atlas (CGGA) and Cancer Genome Atlas (TCGA) databases, identified five lncRNAs through weighted gene coexpression network analysis (WGCNA) to construct Cox regression model, which showed high accuracy in predicting the survival rate of glioma patients.

### Cutaneous Squamous Cell Carcinoma

Cutaneous squamous cell carcinoma is the second most common non melanoma skin cancer, accounting for 20% of skin cancers (71). A variety of LncRNAs have been reported to be abnormally expressed in skin squamous cell carcinoma (72), participating in the complex cancer signal network in skin malignant tumors (73). Studying the signal pathway of tumor development can generate targeted therapy molecules. Liu et al. (44) found that the expression of LINC00641 was significantly reduced in cutaneous squamous cell carcinoma cell lines compared with normal human immortalized keratinocytes. Overexpression of LINC00641 reduced cell proliferation, migration and invasion. *In vivo* experiments also proved that overexpression of LINC00641 inhibited tumor formation in nude mice. Starbase predicted that LINC00641 could target miR-424 in the mechanism research. LINC00641 can be paired with miR-424. Both *in vivo* and *in vitro* experiments showed that the overexpression of miR-424 reversed the inhibitory effect of LINC00641 overexpression on the proliferation, invasion and migration of cutaneous squamous cell carcinoma. Therefore, LINC00641 can inhibit the development of cutaneous squamous cell carcinoma by down regulating miR-424 in cellular and animal studies, which is of great significance for the pathogenesis and therapeutic targets of squamous cell carcinoma.

### Acute Myeloid Leukemia

Acute myeloid leukemia (AML) is the most common acute leukemia in adults, accounting for approximately 80% of the cases in this group, with incidence increasing with age (74). The detection of cytogenetic markers and other molecular markers such as point mutation, epigenetic and proteomic profiles are playing an emerging role in disease risk prediction, diagnosis and prognosis (75). Of course, there are many lncRNAs, which play an important role in hematopoietic cell transformation, disease progression and drug resistance (76). Wang et al. (40) found that the expression of LINC00641 increased in acute myeloid leukemia tissues and cell lines compared with normal tissues and cells. In leukemia cell lines, silencing LINC00641 inhibited the proliferation, migration and invasion of cancer cells, caused cell cycle arrest and induced apoptosis. When exploring the mechanism of LINC00641 in acute myeloid leukemia, LINC00641 was first located in the cytoplasm, and most LncRNAs in the cytoplasm were ceRNA mechanisms. Then, Starbase predicted that LINC00641 had a binding site with miR-378a. It was demonstrated that LINC00641 regulates the malignant biological behavior of leukemia cells through sponge miR-378a. Finally, ZBTB20 was predicted to be the target gene of miR-378a through miRDB, TargetScan and Starbase databases. Cell experiments verified that LINC00641 competitively combined with miR-378a to increase the expression of ZBTB20, thereby affecting the biological behavior of leukemia cells. In conclusion, LINC00641 acts through miR-378a/ZBTB20 signaling pathway and is expected to become a therapeutic target for acute myeloid leukemia.

### Gastric Cancer

Gastric cancer is the fifth most common cancer and the third leading cause of cancer death in the world. The risk factors of the disease include Helicobacter pylori infection, age, high salt intake, etc. (77). The most common tumor markers clinically used for early detection of gastric cancer include CEA, CA19-9 and CA72-4, but the positive rate of these three is about 20-30% (78). Due to low specificity and sensitivity, more and more studies have been conducted on the molecular mechanism of gastric cancer. Among them, lncRNA shows clinical potential in biomarkers, diagnosis and prognosis of gastric cancer (79). Hu et al. (45) found that LINC00641 was highly expressed in 173 gastric cancer tissues compared with adjacent tissues and could be used as a biomarker to predict the overall survival rate of gastric cancer patients. *In vitro* experiments demonstrated that LINC00641 promoted cell proliferation and migration by inhibiting miR-582-5p. Meanwhile, compared with normal gastric cancer cell lines, the expression of LINC00641 in oxaliplatin resistant cell lines was increased, while the expression of miR-582-5p was decreased. When further exploring the mechanism of drug resistance, it was found that after down regulating LINC00641, the expression of LC3II in oxaliplatin resistant cells was inhibited, the expression of LC3 I and p62 was enhanced, and autophagy was inhibited, making gastric cancer cells more sensitive to oxaliplatin. Combined with the down-regulation of miR-582-5p can reverse the above expression, so it is concluded that LINC00641 can regulate oxaliplatin resistance by inhibiting the expression of miR-582-5p and inducing autophagy enhancement. LINC00641 and miR-582-5p participate in the regulation of oxaliplatin resistance by altering autophagy in gastric adenocarcinoma, providing a direction for the treatment of gastric cancer. Hang et al. (38) also found that silencing LINC00641 in gastric cancer cell line reduced the proliferation, migration and invasion of cancer cells and induced apoptosis. After down regulating LINC00641 in cells, the expression of miR-429 increased. Combined with bioinformatics prediction, Notch-1 may be a potential target of miR-429. Finally, the experiment proved that LINC00641 increased the expression of Notch-1 by competitive binding to miR-429 and increased the biological behaviors of gastric cancer cell proliferation, migration and invasion. In conclusion, LINC00641 can function in gastric cancer by targeting miR-429/Notch-1 axis. Due to its carcinogenic function, LINC00641 can be used for future clinical application in the treatment of gastric cancer.

### Colorectal Cancer

Colorectal cancer is the third most common cancer, which causes nearly 700000 deaths annually, with the highest incidence rate in developed countries, and is the fourth most common cause of cancer-related death (80, 81). LncRNAs play an important role in the growth and metastasis of colorectal cancer, especially as competitive endogenous RNAs (ceRNAs), targeting miRNAs to regulate downstream target genes of colorectal cancer (82). Xue et al. (46) found that the expression of LINC00641 in rectal cancer tissues and cells was higher than that in normal rectal tissues and cells. When LINC00641 was knocked down, the proliferation and migration of rectal cancer cells decreased. Bioinformation predicted that miRNA-424-5p was the downstream target of LINC00641, and cell assay proved that the expression of miRNA-424-5p was increased after LINC00641 knockdown. PLSCR4 was predicted to be the downstream target gene of miRNA-424-5p. After knocking down miRNA-424-5p in rectal cancer cells, PLSCR4 expression was up-regulated, and PLSCR4 was positively correlated with LINC00641. In conclusion, LINC00641 competitively binds miRNA-424-5p to relieve the inhibition of PLSCR4. LINC00641 plays a carcinogenic role in rectal cancer through miRNA-424-5p/PLSCR4 axis, which can provide a new target for the treatment of rectal cancer.

### Renal Cell Carcinoma

Globally, renal cell carcinoma is the ninth most common cancer, with rates varying geographically, and the incidence rate of developed countries is the highest (83). To date, there are no screening procedures or reliable biomarkers for the early diagnosis of renal cell carcinoma (84). Datas about lncRNA as a diagnostic biomarker of renal cell carcinoma are limited (85). However, some serum and urine biomarkers have been proposed as potential screening tools. Circulating lncRNA, as an easily accessible blood based biomarker, has been recommended for urogenital malignancies, such as prostate, bladder and kidney cancer (86). Zhang et al. (37) detected 48 cases of renal cell carcinoma and adjacent normal tissues and found that the expression of LINC00641 was increased in renal cell carcinoma, which was related to stage and prognosis. Knockdown of LINC00641 in renal cell carcinoma cell line decreased cell proliferation, invasion and increased apoptosis. It is predicted by Starbase that miR-340-5p can be used as a potential target of LINC00641. Cell experiments demonstrated the interaction between LINC00641 and miR-340-5p, and the expression of miR-340-5p was up-regulated after silencing LINC00641. Inhibition of miR-340-5p could eliminate the pro-apoptotic and anti-invasive effects of LINC00641 silencing on renal cell carcinoma cell line but had no significant effect on cell proliferation. *In vivo* experiments showed that inhibition of LINC00641 reduced tumor volume and increased miR-340-5p expression in nude mice. In conclusion, LINC00641, as a carcinogen of renal cell carcinoma, plays a role by targeting mir-340-5p through ceRNA mechanism, and can be used as a potential target for the treatment of renal cell carcinoma.

### Thyroid Cancer

Thyroid cancer is a common endocrine malignancy tumor. Its incidence rate continues to rise worldwide, and remains one of the lowest mortality rates of human cancers (87). LncRNA is now considered to be an important regulatory molecule involved in the progression of thyroid cancer (88). Rao et al. (89) obtained samples from Co-LncRNA database, including 12 normal samples and 83 thyroid cancer samples, and identified differentially expressed LncRNAs by linear model of microarray analysis (limma), and finally identified 6 up-regulated and 85 down-regulated lncrnas. It is predicted that LINC00641 is down-regulated in thyroid cancer and is associated with longer disease-free survival, but it is still lack of experimental verification.

## Clinical Significance of LINC00641

Serum cancer biomarkers are limited and nonspecific, with limitations (90). Therefore, exploring more targets will contribute to the early diagnosis, treatment and prognosis of cancer. The mechanism of LINC00641 in cancer has been concentrated in recent 5 years, which can be used as an emerging biomarker of cancer prognosis.

### As a Biomarker of Cancer Prognosis

Li (35) divided 39 bladder cancer tissues into LINC00641 high group and LINC00641 low group according to the median LINC00641 level. Kaplan-Meier curve analysis showed that low expression of LINC00641 was associated with reduced overall survival and progression free survival. Mao (42) analyzed the expression of LINC00641 in 166 breast cancer tissues, and statistical analysis showed that LINC00641 was significantly correlated with tumor size, lymph node metastasis and clinical stage (P < 0.01). Liu (36) analyzed 23 prostate cancer patients and found that the low LINC00641 expression group had a lower survival rate than the high LINC00641 expression group (P < 0.05). Hu (38) detected the expression of LINC00641 in 173 cases of gastric cancer, which was related to M stage (P < 0.01). Cox regression model including LINC00641, miR-582-5p, CEA and age could predict the 1-year and 3-year survival rates of gastric cancer patients (C index was 0.876 ± 0.06). Xue’s study (46) included 50 cases of rectal cancer and made a survival curve, and found that the high LINC00641 expression group had worse survival (P < 0.05). Zhang (37) analyzed 48 cases of renal cell carcinoma and found that the expression of LINC00641 was related to clinical T stage (P < 0.01) and metastasis (P < 0.05), and Kaplan-Meier survival analysis showed that the high LINC00641 expression group had poor prognosis (P < 0.05). In conclusion, the differential expression of LINC00641 is related to survival time and can be used as a biomarker of cancer prognosis, such as combining with known molecular biomarkers of cancer, to increase the reliability of predictive value.

### As a Target for Cancer Treatment

As a tumor suppressor gene: the over expression of LINC00641 in bladder cancer cell lines reduces cell proliferation, migration and invasion ability (35). *In vivo*, LINC00641 overexpression significantly inhibited tumor volume. In cervical cancer cell lines, up-regulation of LINC00641 inhibits cell proliferation, increases apoptosis, reduces invasion and migration ability, and inhibits EMT (41). In breast cancer cell lines, overexpression of LINC00641 inhibits cell proliferation, migration and invasion, stagnates cells in G1 phase and promotes apoptosis (42). In prostate cancer cell lines, over expression of LINC00641 inhibits cell proliferation, invasion and promotes apoptosis (36). In non-small cell lung cancer cell lines, overexpression of LINC00641 reduces the ability of proliferation and migration and increases apoptosis (39). Overexpression of LINC00641 in glioma cells inhibits cell proliferation and promotes apoptosis (43). In cutaneous squamous cell carcinoma, LINC00641 overexpression decreased cell proliferation, migration and invasion ability *in vitro*, and the tumor volume of LINC00641 overexpression group was smaller in nude mouse tumorigenesis experiment (44).

As an oncogene: in leukemia cell lines, silencing LINC00641 inhibits the proliferation, migration and invasion of cancer cells, causes cell cycle arrest and induces apoptosis (40). Silencing LINC00641 reduces the proliferation, migration and invasion of cancer cells and induces apoptosis in gastric cancer cell lines (38), and LINC00641 participates in regulating oxaliplatin resistance by changing autophagy in gastric adenocarcinoma, providing a direction for the treatment of gastric cancer (45). When LINC00641 was knocked down, the proliferation and migration of rectal cancer cells decreased (46). Knockdown LINC00641 in renal cell carcinoma cell line decreased cell proliferation, invasion and increased apoptosis (37). Therefore, LINC00641 can change the sensitivity of tumor to drugs by regulating the biological behavior of tumor cell proliferation and invasion, and it is a promising target for cancer treatment.

Many LncRNAs have been shown to be potential biomarkers and targets for cancer diagnosis and treatment (91). The expression characteristics and clinical significance of LncRNA in tumors have been proved by *in vivo* and *in vitro* experiments, but it still needs some time to be applied in clinical practice. One of the difficulties is that it is currently impossible to determine the sequences and structural elements that allow long-chain non-coding RNA molecules to perform their cellular functions (92). Secondly, new effective and stable genome editing strategies, as well as more effective and less toxic gene therapy delivery systems, need to be developed before LncRNAs can become potential therapies (80). Moreover, the expression of LncRNA is usually low, which increases the difficulty of detection. Although a variety of methods for detecting cancer biomarkers have been created, most of them are only conceptual demonstrations, which benefit from highly optimized laboratory conditions (93). However, with the rapid improvement of experimental technology, exploring LncRNA as a biomarker of cancer is of great significance to clarify the diagnosis of cancer and improve the prognosis of cancer.

## Conclusions, Limitations and Future Prospects

**Figure 4:**
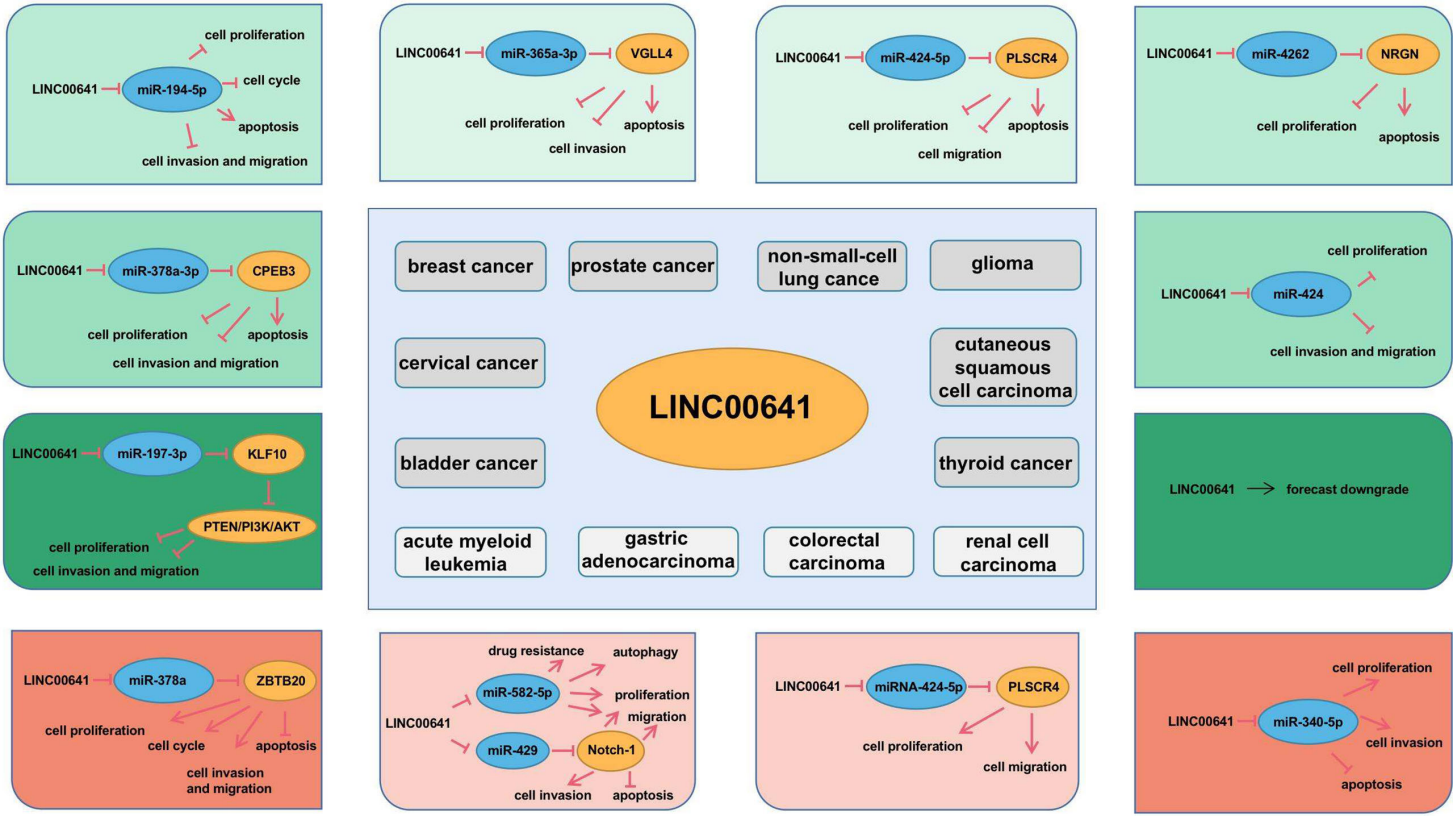
Molecular mechanism of LINC00641 in different human cancers.

The mechanism of LINC00641 in different tumors has been summarized above. Current studies focus on the ceRNA mechanism. LINC00641 was predicted in the database to be 83% localized in the cytoplasm, conforming to the localization of ceRNA mechanism. Through the database prediction and verification in cell experiments, LINC00641 can be used as miR-197-3p、miR-378a-3p、miR-194-5p、miR-365a-3p、miR-424-5p、miR-4262、miR-424、miR-378a、miR-582-5p、miR-429、miRNA-424-5p、microRNA-340-5p sponges (**Figure 4**). LINC00641 is involved in tumorigenesis in more than one way, so it may be difficult to use as a therapeutic target for different tissues. In a review of the mechanism of lncRNA in the cytoplasm, ceRNA has been studied the most. LncRNA mediated ceRNA network plays a key role in the proliferation, invasion, apoptosis and autophagy of varieties of tumors, as well as distant metastasis, epithelial mesenchymal transformation and chemoresistance. The inclusion of abnormally expressed lncRNA, miRNA and mRNA in tumors and the construction of ceRNA network help to clarify the regulatory mechanism of lncRNA as ceRNA to promote tumorigenesis, and further evaluate the potential therapeutic targets and prognostic biomarkers of cancer (82, 94–96). At present, there is still much room for exploring the research mechanism of LINC00641. For example, LncRNA containing Alu element can mediate mRNA decay in the cytoplasm by recruiting STAU1 protein (97), and LncRNA can also act as RBP molecular bait involved in mRNA decay. For example, lncRNA OCC-1 can regulate the level of a large number of mRNA at the post transcriptional level by regulating the stability of RBP HuR (98). Secondly, LncRNA can also regulate translation or post-translational modification in the cytoplasm. For example, lincRNA-p21 plays a post transcriptional function as a translation regulator (99). Post translational modification mediated by LncRNA is the hub of many cellular signaling pathways. LncRNA can act on post-translational modification of metabolic enzymes, transcription factors or other proteins involved in energy metabolism pathways, including ubiquitination and acetylation, which provides a new idea for cancer energy metabolism (100). In addition to the above mechanism of action in the cytoplasm, although LINC00641 does not account for a high proportion in the nucleus, the role of LINC00641 in the nucleus can be further explored. Such a large number of long chain non-coding RNAs can interact with genes, non-coding genes and proteins to build a crisscross network in the abnormal regulation of cancer.

The above study of LINC00641 was performed in tissue cells. It is an invasive operation to detect the gene expression level in solid tumors, which needs to be obtained from surgical or biopsy specimens. Liquid biopsy, which detects cancer genes from blood or body fluids, has attracted more and more attention (101). LncRNA can be secreted out of cells by exosomes and regulate the occurrence and progression of cancer by changing the tumor microenvironment (102). Exosome mediated LncRNA can regulate malignant biological behaviors such as tumor apoptosis, invasion and migration, promote lymph node metastasis of cancer and affect the drug resistance of tumor drugs (103–106). In conclusion, exosomal LncRNA can be regarded as a novel biomarker of cancer progression and a potential therapeutic target for clinical application (107).

At present, the research data on LINC00641 in tumors are limited. In view of the numerous mechanisms of LncRNA, we look forward to more research on LINC00641 in tumors, enrich the significance of LINC00641 and contribute to its early clinical application.

## Author Contributions

Conceptualization: XH and SZ. Methodology: SZ. Data curation: XH and SZ. Writing—original draft preparation: XH. Writing—review and editing: XH and SZ. All authors contributed to the article and approved the submitted version.

## Conflict of Interest

The authors declare that the research was conducted in the absence of any commercial or financial relationships that could be construed as a potential conflict of interest.

## Publisher’s Note

All claims expressed in this article are solely those of the authors and do not necessarily represent those of their affiliated organizations, or those of the publisher, the editors and the reviewers. Any product that may be evaluated in this article, or claim that may be made by its manufacturer, is not guaranteed or endorsed by the publisher.
